# Data in support of transcriptional regulation and function of Fas-antisense long noncoding RNA during human erythropoiesis

**DOI:** 10.1016/j.dib.2016.03.106

**Published:** 2016-04-07

**Authors:** Olga Villamizar, Christopher B. Chambers, Yin-Yuan Mo, Donald S. Torry, Reese Hofstrand, Janice M. Riberdy, Derek A. Persons, Andrew Wilber

**Affiliations:** a Department of Medical Microbiology, Immunology and Cell Biology, Southern Illinois University School of Medicine, Springfield, IL, USA; b Simmons Cancer Institute, Springfield, IL, USA; c Department of Pharmacology and Toxicology and Cancer Institute, University of Mississippi Medical Center, Jackson, MS, USA; d Department of Hematology, St. Jude Children׳s Research Hospital, Memphis, TN, USA

**Keywords:** Erythropoiesis, Long noncoding RNA, Saf, Apoptosis, Fas

## Abstract

This paper describes data related to a research article titled, “Fas-antisense long noncoding RNA is differentially expressed during maturation of human erythrocytes and confers resistance to Fas-mediated cell death” [1]. Long noncoding RNAs (lncRNAs) are increasingly appreciated for their capacity to regulate many steps of gene expression. While recent studies suggest that many lncRNAs are functional, the scope of their actions throughout human biology is largely undefined including human red blood cell development (erythropoiesis). Here we include expression data for 82 lncRNAs during early, intermediate and late stages of human erythropoiesis using a commercial qPCR Array. From these data, we identified lncRNA Fas-antisense 1 (Fas-AS1 or Saf) described in the research article. Also included are 5′ untranslated sequences (UTR) for lncRNA Saf with transcription factor target sequences identified. Quantitative RT-PCR data demonstrate relative levels of critical erythroid transcription factors, GATA-1 and KLF1, in K562 human erythroleukemia cells and maturing erythroblasts derived from human CD34^+^ cells. End point and quantitative RT-PCR data for cDNA prepared using random hexamers versus oligo(dT)_18_ revealed that lncRNA Saf is not effectively polyadenylated. Finally, we include flow cytometry histograms demonstrating Fas levels on maturing erythroblasts derived from human CD34^+^ cells transduced using mock conditions or with lentivirus particles encoding for Saf.

## **Specifications table**

**Table:** 

Subject area	*Cell Biology, Biochemistry*
More specific subject area	*Erythropoiesis, transcription, apoptosis*
Type of data	*Table and Figures*
How data was acquired	*Quantitative RT-PCR (StepOnePlus Thermocycler, Applied Biosystems)*
*Flow cytometry (FACSAriaII, BD Biosciences) using FlowJo v10.0 analysis software.*
Data format	*Processed/Analyzed data*
Experimental factors	*Isolation of total cellular RNA, cDNA amplification, PCR analysis, antibody staining of cells*
Experimental features	*Analysis of gene expression by quantitative RT-PCR and flow cytometry*
Data source location	*Springfield, IL, United States*
Data accessibility	*Data is with this article.*

## **Value of the data**

• Expression data for 82 long noncoding RNAs at early and late stages of human erythroid maturation are provided.
• Methods to determine poly-adenylation status of lncRNAs using end point and quantitative RT-PCR analysis of cDNA.
• Methods to transduce human CD34^+^ cells with lentiviral vectors encoding for expression of a functional lncRNA and subsequently monitor Fas receptor levels by flow cytometry.

## 1 Data

**Fig. 1:**
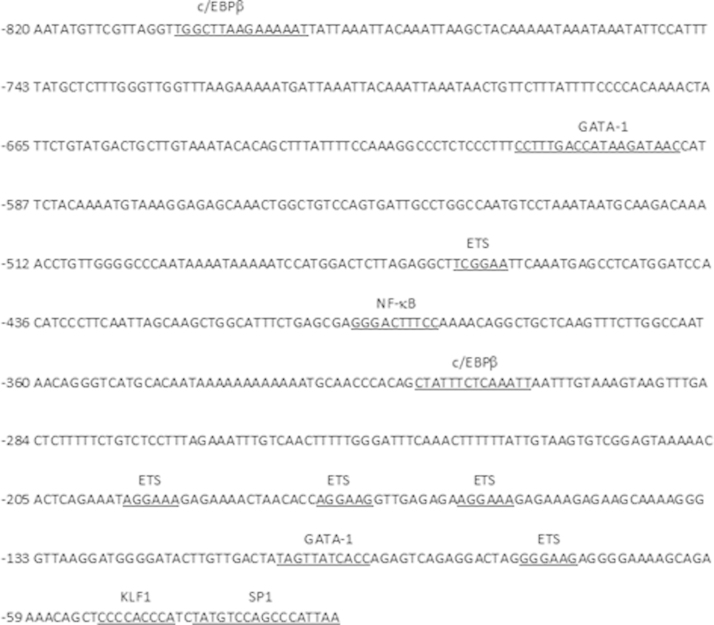
Saf 5′ untranslated region and transcription factor binding sites. Saf promoter sequence (nucleotides −820 to –23 bp relative to Saf transcriptional start site) used in luciferase reporter assays. Underlined sequences are binding sites for GATA-1, KLF1, NF-κB, SP1, ETS, and c/EBPβ (CCAAT-enhancer binding protein).

**Fig. 2:**
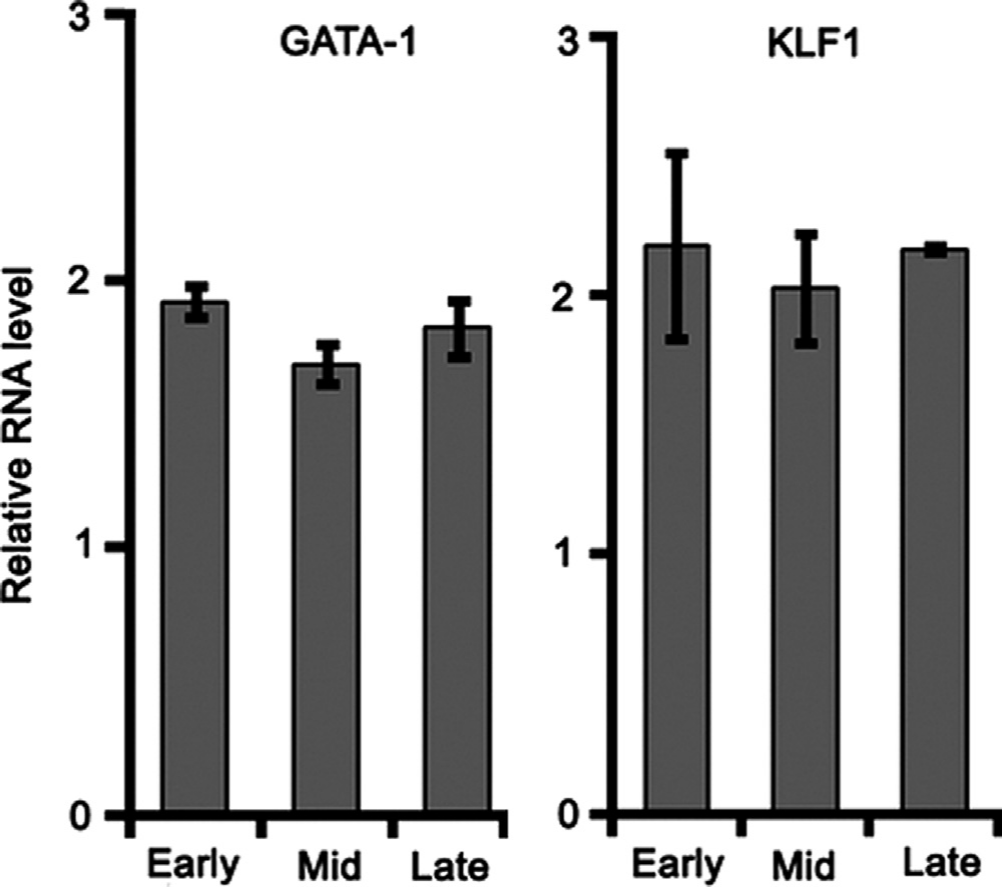
Transcript levels of GATA-1 and KLF1 in maturing erythroid cells derived from CD34^+^ cells of independent ontology. Erythroid cells derived from fetal liver or adult bone marrow CD34^+^ cells exposed to identical conditions were collected during early, intermediate (mid), or late stages of differentiation. Cells were isolated of total RNA and levels of GATA-1 and KLF1 transcripts determined by quantitative RT-PCR. Data were normalized to RNAseP, set relative to fetal liver, and plotted as mean+SD; *n*=2 independent donors performed in duplicate.

**Fig. 3:**
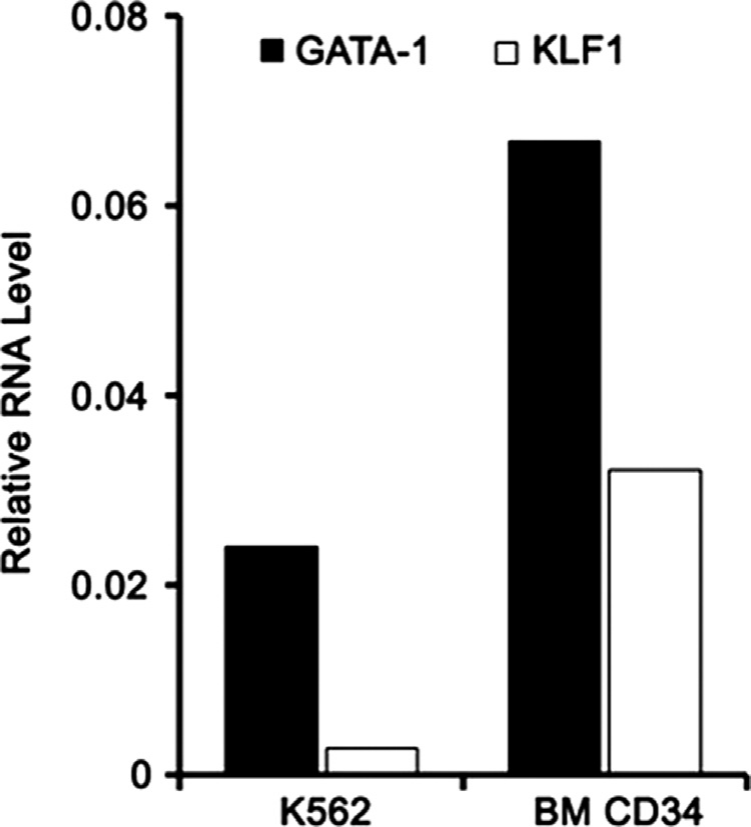
GATA-1 and KLF1 expression in K562 cells and adult bone marrow-derived erythroid cells. Relative transcript levels of GATA-1 and KLF1 for K562 human erythroleukemia cells and adult bone marrow (BM CD34)-derived erythroblasts collected at late stage of culture. Data were normalized to RNAseP and mean levels plotted.

**Fig. 4:**
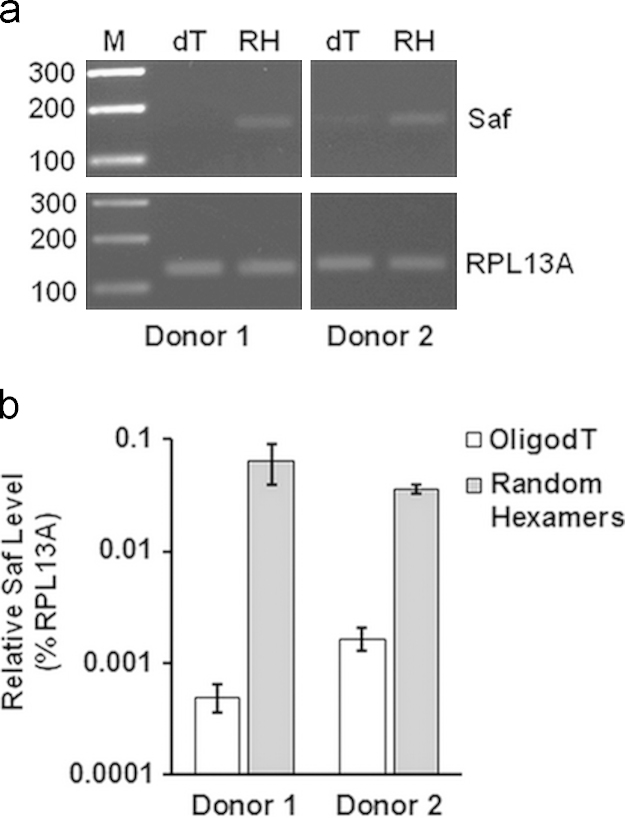
LncRNA Saf is not effectively polyadenylated. Total RNA was isolated from erythroblasts-derived from CD34^+^ cells on culture day 8 and reverse transcribed into cDNA using oligo(dT)_18_ or random hexamers. (A) End point RT-PCR of Saf lncRNA and RPL13A for cDNA produced with oligo(dT)_18_ (dT) or random hexamers (RH). M, 100-bp ladder. Vertical white lines have been inserted to represent repositioned lanes on gel images. (B) Real time quantitative RT-PCR analysis of Saf lncRNA for cDNA prepared using the indicate primers. Data were normalized to RPL13A and plotted as mean+SD; *n*=2 independent donors performed in triplicate.

**Fig. 5:**
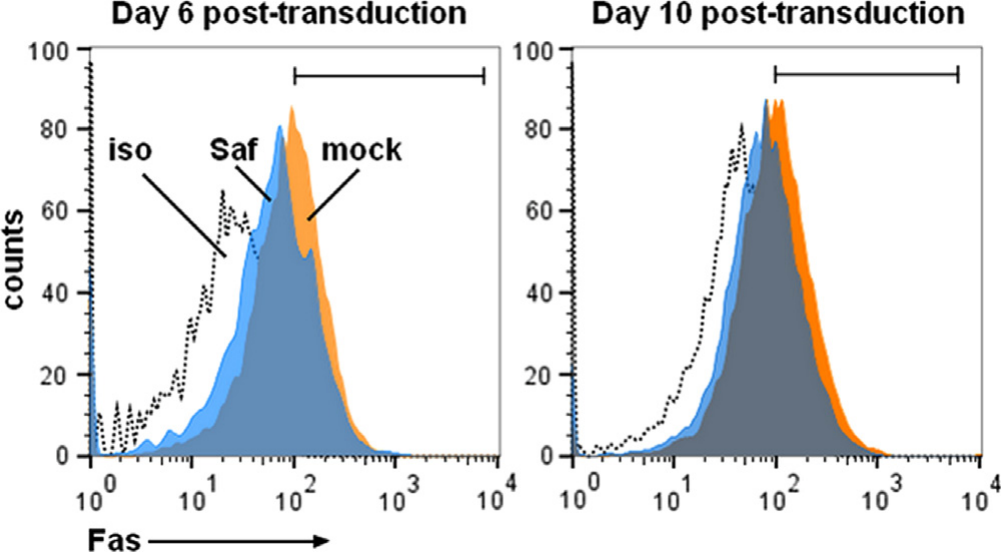
LncRNA Saf overexpression in erythroblasts reduces surface expression of Fas. Human CD34^+^ cells from a single healthy donor were transduced using mock conditions or with lentiviral vector particles encoding for Saf. Flow cytometry histograms demonstrating surface levels of Fas receptor for mock (orange) and Saf (blue) transduced cells collected on days 6 and 10 post-transduction, times corresponding to proliferation and differentiation stages of culture. Dotted line depicts cells reacted with isotype control antibodies.

Here we provide expression data for a focused set of lncRNAs during culture-induced differentiation of human CD34^+^ cells into erythroblasts (Table 1). We provide sequences for the cloned 5′ untranslated region (UTR) of Saf highlighting transcription factor binding sites including GATA-1, KLF1, and NF-κB, which were studied in detail in the research article (Fig. 1). We include relative transcript levels of GATA-1 and KLF1 in K562 human erythroleukemia cells and maturing human erythroblasts (Fig. 2, Fig. 3). We share end point and quantitative RT-PCR analysis of cDNA prepared from total RNA isolated from human erythroblasts using random hexamers versus oligo(dT)_18_ (Fig. 4). Finally, we include flow cytometry histograms demonstrating Fas surface levels on maturing erythroblasts derived from human CD34^+^ cells transduced using mock conditions or Saf-encoding lentivirus particles (Fig. 5).

## 2 Experimental design, materials and methods

### 2.1 LncRNA quantitative RT-PCR array

CD34^+^ cells isolated from fetal liver (FL), umbilical cord blood (CB), or adult bone marrow (BM) were purchased commercially (Lonza; Walkersville, MD) and used in accordance with protocols approved by the Springfield Committee for Research Involving Human Subjects at Southern Illinois University School of Medicine. Samples for FL and BM were from two unrelated donors. CD34^+^ cells were cultured under conditions that promote erythroid differentiation as described [2]. Erythrocytes collected at early, intermediate and late stages of maturation were extracted of total RNA using spin columns (Ambion) with on-column DNase treatment (Promega). RNA was quantified by a Nanodrop 2000 (Life Technologies) and quality assessed by visualizing 18S and 28S ribosomal RNA bands separated through 1% agarose and stained with ethidium bromide. RNA (300 ng) was reverse transcribed into cDNA using SuperScript VILO Master Mix (Life Technologies) and conditions: 25 °C–10 min, 42 °C–60 min, 85 °C–5 min, 4 °C-hold. cDNA (40 ng/reaction) was used as template for a lncRNA PCR array (LncRNA Profiler™ qPCR Array, System Biosciences) that included primer sequences for 82 unique lncRNAs and 14 controls optimized for use with SYBR Green as the fluorophore. A version of this array has previously been validated [3]. Quantitative PCR reactions were performed with a StepOnePlus thermocycler (Applied Biosystems) using SYBR Green settings that included a melt curve. Changes in transcript levels were assessed by the delta-delta C_T_ formula [4] with normalization to internal controls.

### 2.2 In silico analysis of Saf 5′ untranslated region (UTR)

We performed in silico analysis of genomic DNA within 1000-bp upstream of the Saf transcriptional start site. Transcription factor binding sites were identified using Geneious (v6.1.2, Biomatters Ltd.) and UCSC Genome Browser on Human Dec. 2013 (GRCh38/hg38) assembly (http://genome.ucsc.edu/) [5].

### 2.3 Relative expression of GATA-1 and KLF1

RNA, extracted from K562 human erythroleukemia cells or culture-differentiated human erythroblasts collected at early, intermediate and late stages of maturation, was reverse transcribed into cDNA as described in Section 2.1. Quantitative PCR was performed on a StepOnePlus thermocycler (Applied Biosystems) using cDNA (100 ng) and TaqMan primer:probe sets specific to GATA-1 (Hs01085823_m1; FAM), KLF1 (Hs00610592_m1; FAM), and RNaseP (4316844; VIC-TAMRA) all from Life Technologies. Changes in transcript levels were assessed by the delta-delta C_T_ formula with normalization to RNAseP.

### 2.4 Saf polyadenylation status

RNA was extracted from CD34^+^-derived erythroblasts on day 8 in culture and subjected to first strand cDNA synthesis with either random hexamers or oligo(dT)_18_ according to the manufacturer׳s protocol (ThermoScientific). Resulting cDNAs (100 ng) were quantified with SYBR Green by real time PCR (StepOnePlus, Life Technologies) and analyzed by end point PCR using Saf primers (For: 5′-ATG TGA ACA TGG AAT CAT CAA GG-3′; Rev: 5′-GGA GAT TCA TGA GAA CCT TGG-3′) and RPL13A primers (For: 5′-CCT GGA GGA GAA GAG GAA AGA GA-3′; Rev: 5′ TTG AGG ACC TCT GTG TAT TTG TCA A-3′) as an internal control. End point PCR products were resolved on 2% agarose gels containing ethidium bromide and photographed.

### 2.5 Flow cytometry analysis of Fas on maturing erythroblasts

Flow cytometry studies were performed on a FACSAriaII (BD Biosciences) using FlowJo v10.0 analysis software. Human CD34^+^ cells transduced using mock conditions or with lentiviral vector particles encoding for Saf were collected on days 6 and 10 post-transduction, times corresponding to proliferation and differentiation stages of culture. Erythroblasts (1×10^5^) were washed with PBS, suspended in staining medium (PBS+0.5% BSA) and reacted with allophycocyanin (APC)-conjugated mouse anti-human CD95 (DX2, BD Biosciences, 1:50 dilution); mouse anti-human IgG-APC was used as an isotype control.

### 2.6 Statistical analysis

Microsoft Excel or Prism 5 (GraphPad) were used to determine descriptive statistics (mean+SD).

## Figures and Tables

**Table 1:** Expression of lncRNAs during erythroid maturation.

**lncRNA**	**Fold change day 6**	**Fold change day 10**
21A	0.71	1.45
AAA1	3.00	ND
aHIF	1.42	1.41
Ak023948*	**0.50**	**0.33**
ANRIL	5.66	ND
Anti-NOS2A**	**2.83**	**10.49**
BACE1AS	0.72	2.80
BC017743	5.68	ND
BC043430	2.76	ND
BC200	0.71	0.72
BCMS	0.71	1.42
Bic	1.39	0.71
CCND1 ANCR	ND	ND
CMPD	12.47	ND
DD3	5.67	ND
DGCR5	ND	ND
DISC2	2.85	1.32
DLG2AS	11.40	ND
Ego	2.85	1.32
Gas5	0.71	2.82
Gomafu**	**2.84**	**11.36**
H19*	**0.02**	**0.09**
H19-AS	ND	ND
Har1a	ND	ND
Har1b	ND	2.96
Hotair	12.14	ND
HotairM1*	**0.36**	**0.36**
HOTTIP	6.05	ND
Hoxa11as BE305073	ND	ND
Hoxa1as AA489505	1.43	0.71
Hoxa3as BE873349	ND	ND
Hoxa3as BI823151	3.05	ND
Hoxa6as AK092154	1.51	ND
HULC	ND	ND
I1pa16	11.37	ND
IGF2AS	ND	ND
IPW	1.41	0.71
KRASP1	1.43	2.79
lincRNA-SFMBT2	ND	ND
lincRNA-VLDLR	11.32	ND
LIT	1.40	1.43
Loc285194	2.88	ND
LUST	0.72	2.81
Malat1**	**2.43**	**5.65**
MEG3	ND	ND
MER11c	2.85	ND
NCRMS	ND	ND
NDM29	5.69	ND
NEAT1	0.71	5.63
PAR1	2.81	ND
PAR5	0.71	0.71
PCAT-1	11.33	ND
PCAT-14	6.07	ND
PCAT-29	ND	ND
PCAT-32	11.45	ND
PCAT-43	1.47	ND
PCGEM1	1.52	ND
PR-AT2	1.43	2.81
PRINS	ND	ND
PSF inhibiting RNA	5.67	ND
PtenP1	0.71	1.41
RMRP	0.71	2.83
ROR	6.02	ND
Saf**	**2.83**	**5.68**
SCA8	2.86	ND
Sox2OT	11.38	ND
SRA	1.42	0.71
ST7OT1	1.42	2.82
ST7OT2	ND	ND
ST7OT3	ND	ND
ST7OT4	1.42	2.84
telomerase RNA	0.72	2.82
Tmevpg1	6.09	ND
TU_001762 9	3.03	ND
TUG1	0.71	1.42
UCA1	ND	ND
WT-1as	11.38	ND
Y1**	**2.42**	**5.58**
Y3	1.41	2.83
Y4	1.41	1.41
Y5	1.42	0.71
Zeb2NAT	0.71	1.34

In bold are lncRNAs with a 2-fold *decrease or **increase on both days 6 and 10; ND, not detected.
